# Oxidative stress response and NRF2 signaling pathway in autism spectrum disorder

**DOI:** 10.1016/j.redox.2025.103661

**Published:** 2025-05-02

**Authors:** Sergio Davinelli, Alessandro Medoro, Martina Siracusano, Rosa Savino, Luciano Saso, Giovanni Scapagnini, Luigi Mazzone

**Affiliations:** a Department of Medicine and Health Sciences "V. Tiberio", University of Molise, Campobasso, Italy; b Department of Biomedicine and Prevention, University of Rome Tor Vergata, Rome, Italy; c Child Neurology and Psychiatry Unit, Department of Wellbeing of Mental and Neurological, Dental and Sensory Organ Health, Policlinico Tor Vergata Hospital, Rome, Italy; d Department of Clinical and Experimental Medicine, University of Foggia, Foggia, Italy; e Department of Physiology and Pharmacology “Vittorio Erspamer”, Sapienza University of Rome, Rome, Italy

**Keywords:** NRF2, Autism, Oxidative stress, Inflammation, Antioxidants

## Abstract

The prevalence of autism spectrum disorder (ASD), a neurodevelopmental disorder characterized by impairments in social communication and restricted/repetitive behavioral patterns, has increased significantly over the past few decades. The etiology of ASD involves a highly complex interplay of genetic, neurobiological, and environmental factors, contributing to significant heterogeneity in its clinical phenotype. In the evolving landscape of ASD research, increasing evidence suggests that oxidative stress, resulting from both intrinsic and extrinsic factors, may be a crucial pathophysiological driver in ASD, influencing neurodevelopmental processes that underlie behavioral abnormalities. Elevated levels of oxidative stress biomarkers, including lipid peroxides, protein oxidation products, and DNA damage markers, alongside deficient antioxidant enzyme activity, have been consistently linked to ASD. This may be attributed to dysregulated activity of nuclear factor erythroid 2-related factor 2 (NRF2), a pivotal transcription factor that maintains cellular redox homeostasis by orchestrating the expression of genes involved in antioxidant defenses. Here, we summarize the converging evidence that redox imbalance in ASD may result from NRF2 dysregulation, leading to reduced expression of its target genes. We also highlight the most promising antioxidant compounds under investigation, which may restore NRF2 activity and ameliorate ASD behavioral symptoms.

## Highlights

• Autism Spectrum Disorder (ASD) is a multifactorial condition caused by both genetic and non-genetic factors.
• Oxidative stress may contribute to ASD by affecting neurodevelopmental processes.
• ASD is linked to high oxidative markers and impaired antioxidant defences.
• NRF2 dysregulation may underlie redox imbalance observed in ASD.
• Antioxidant compounds targeting NRF2 show therapeutic potential in ASD.

## 1 Introduction

According to the recent Global Burden of Disease Study 2021, autism spectrum disorder (ASD) affects approximately 61.8 million individuals worldwide, which translates to about 1 in every 127 people globally. Although this burden diminishes with increasing age, ASD was ranked within the top-ten causes of non-fatal health burden for people younger than 20 years [1]. Phenotypically, ASD is a heterogeneous neurodevelopmental disorder characterized by a core set of symptoms, which include alterations in social interaction, deficits in communication, and the presence of restricted and repetitive behaviors, comprising sensory sensitivities [2]. Beyond core symptoms, individuals with ASD may exhibit challenges in emotional regulation, cognitive and adaptive variability, which can range from intellectual impairment (requiring a high level of support needs) to high cognitive skills (requiring a low level of support needs) [3,4]. Although the exact etiology of the disorder remains unclear, the diverse clinical manifestations of ASD result from a complex interplay of genetic, neurobiological, and environmental factors [5]. A number of genetic alterations, such as copy number variants (CNVs) and single nucleotide polymorphisms (SNP), may affect brain development processes, including cell adhesion, neural development, and synaptogenesis [6]. Likewise, several signaling pathways, including phosphoinositide 3-kinase (PI3K)/Akt, mechanistic target of rapamycin (mTOR), and Wingless/Integrated (Wnt) signaling pathways, are frequently altered in various fetal and early postnatal stages of ASD phenotypes [7]. These alterations can dysregulate neuronal differentiation and growth, lead to increased synthesis of inflammatory cytokines, and cause changes in dendritic spine density and synaptic pruning [8]. Current evidence also suggests that there are several prenatal and perinatal environmental risk factors for ASD, including advanced parental age, maternal health conditions, birth complications, and prenatal chemical exposures [9]. This array of environmental risk factors may interact with genetic predispositions, modifying neurodevelopmental trajectories through several mechanisms, including immune dysregulation, mitochondrial dysfunction, hormonal disruptions, gut microbiome alterations, and oxidative stress [[10], [11], [12]].

Recently, a number of studies have increasingly highlighted the significance of oxidative stress in the pathophysiology of ASD, suggesting an imbalance between the production of reactive oxygen species (ROS) and antioxidant defenses in both the periphery and the brain [13]. Notably, elevated levels of reactive species result in genotoxicity and trigger a sterile inflammatory response, as well as microglial activation in the brain, contributing to the development of ASD. Indeed, redox imbalance has been consistently observed in children with ASD and appears to be associated with decreased plasma levels of reduced glutathione (GSH), a key endogenous antioxidant, and a lower ratio of GSH to oxidized glutathione (GSSG) [[14], [15], [16]]. However, GSH levels are also significantly reduced in the cerebellum and temporal regions of the brain in individuals with ASD. This decline in GSH contributes to oxidative stress, neuroinflammation, and mitochondrial dysfunction, as indicated by increased levels of nitrosative and oxidative damage markers, including 3-nitrotyrosine (3-NT) and 8-oxo-deoxyguanosine (8-oxodG), elevated levels of 3-chlorotyrosine (3-CT), a recognized biomarker of chronic inflammation, and altered aconitase activity, a marker of mitochondrial superoxide production [16,17]. In addition to GSH, other endogenous antioxidants are altered in children with ASD, such as superoxide dismutase (SOD) and catalase (CAT), resulting in extensive oxidative damage to macromolecules, chronic inflammation, and abnormal neuronal cell function [18,19]. The dysregulation of the thioredoxin (Trx) system, a crucial component of cellular redox regulation, has also been linked to ASD, and its dysfunction may further compromise the ability to manage oxidative stress effectively [20].

A battery of cytoprotective genes encoding antioxidant enzymes is transcriptionally activated by nuclear factor erythroid 2-related factor 2 (NRF2) in response to oxidative/genotoxic stress. NRF2 is the major transcription factor sensitive to shifts in the redox status and coordinates the expression of a vast array of genes containing a specific motif (5′-A/GTGAC//nnnGCA/G-3′) in the promoter region that binds NRF2, known as the antioxidant response elements (AREs) [21]. Accordingly, ARE-driven genes, such as *SOD*, heme oxygenase 1 (*HO-1*), glutathione peroxidase 2 (*GPX2*), glutamate-cysteine ligase (*GCL*), glutathione reductase (*GR*), thioredoxin reductase (*TXNRD*), and peroxiredoxins (*PRXs*), along with phase II xenobiotic detoxification enzymes, including NAD(P)H:quinone dehydrogenase 1 (*NQO1*), glutathione-S-transferases (*GSTs*), and UDP-glucuronosyltransferases (*UGTs*), are induced by NRF2 and alleviate oxidative stress and genotoxicity caused by reactive species and environmental factors (e.g., xenobiotics) [22]. Given the association of redox imbalance with several neuropsychiatric disorders and the key role of the NRF2 pathway in counteracting oxidative stress, numerous studies continue to reveal the significant involvement of NRF2 in conditions such as major depressive disorder, bipolar disorder, schizophrenia, and attention deficit hyperactivity disorder (ADHD) [23,24].

In the following sections, we first highlight the relevance of oxidative stress in ASD by examining how dysregulation of redox balance and alterations in oxidative stress biomarkers contribute to its pathophysiology. Next, we discuss recent developments concerning NRF2 in ASD, covering mechanistic aspects and summarizing the contributions of NRF2 and its target genes to the disorder. Finally, the article examines potential compounds that modulate NRF2 by enhancing the activity of its associated enzymes and that are currently under investigation.

## 2 Oxidative stress and ASD

ASD has been increasingly associated with disruptions in redox homeostasis, where excessive ROS generation combined with impaired antioxidant defenses (i.e., oxidative stress), contributes to neuronal dysfunction and neuroinflammation [25,26]. As pleiotropic physiological signaling agents, ROS, particularly hydrogen peroxide (H_2_O_2_), regulate multiple molecular targets and cellular processes, including proliferation, differentiation, and adaptive responses to environmental stress (e.g., antioxidant defense). At low physiological levels, H_2_O_2_ acts as a major signaling molecule by reversibly oxidizing specific protein targets, which modulates their activity, localization, and interactions [[27], [28], [29]]. Unlike other ROS, H_2_O_2_ is much more stable, enabling it to support controlled signaling rather than causing widespread damage. Its ability to cross biological membranes allows it to influence processes at sites distant from where it is produced. H_2_O_2_ reacts mainly with cysteine residues in proteins, which supports the regulation of signaling pathways, such as NRF2 activity, without triggering the immediate and extensive damage characteristic of more reactive species (e.g., hydroxyl radicals). In addition, enzymatic redox systems regulate H_2_O_2_ levels under normal conditions to maintain its signaling role within a safe and effective range [[30], [31], [32], [33], [34]].

However, it should be noted that ROS is an umbrella term for oxygen-containing molecules, including free radicals (e.g., superoxide, hydroxyl, peroxyl) and non-radical oxidants (e.g., hydrogen peroxide, ozone, singlet oxygen), with distinct chemical reactivity that modulates their biological impact. Nevertheless, the pleiotropic actions of ROS, which are beneficial at low physiological concentrations (i.e., promoting cell proliferation, differentiation, and migration), may become detrimental under conditions characterized by increased ROS levels [29]. The antagonistic pleiotropic effect of ROS is particularly relevant in the embryonic nervous system, where ROS influence neuronal polarity, neuronal connectivity, synaptic transmission, and neuronal network organization [35]. For instance, it has been reported that H_2_O_2_, depending on its physiological concentration, may contribute to promoting axonal and neuronal growth. Conversely, a pathological increase in ROS levels, including H_2_O_2_, has been associated with growth cone collapse and axonal degeneration [36]. In this context, maternal immune activation (MIA), a significant environmental risk factor linked to the development of ASD, has been reported to upregulate the expression of ROS-producing enzymes in the fetal brain, including NADPH oxidases (NOXs), the major endogenous enzymatic sources of superoxide anion radical (O_2_·^-^) and H_2_O_2_, leading to the loss of Purkinje cells and the development of ASD-like behaviors [37]. Increased expression of NOXs, particularly the NOX2 isoform, has also been observed in B cells from children with ASD [38].

**Fig. 1:**
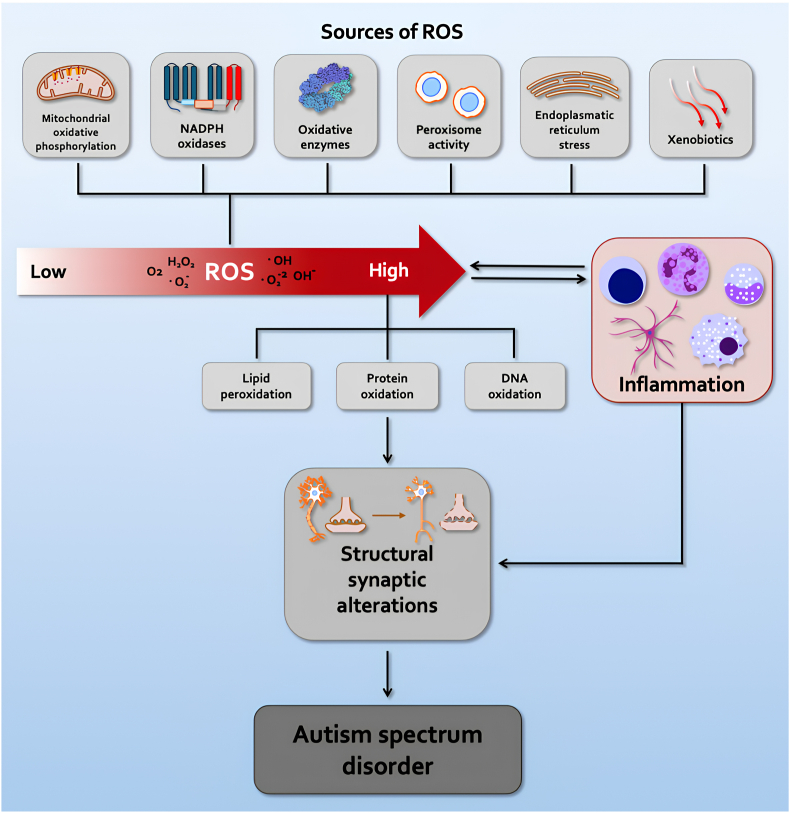
**Oxidative stress in the context of autism spectrum disorder**. The figure depicts a shift from physiological ROS levels, essential for normal redox signaling, to excessive concentrations that promote cellular damage. Endogenous sources of ROS include mitochondrial oxidative phosphorylation, NADPH oxidases, oxidative enzymes, peroxisomal activity, and endoplasmic reticulum stress, alongside environmental factors, such as xenobiotic exposure. Increased levels of ROS, such as hydrogen peroxide (H_2_O_2_), superoxide (O_2_•^-^), and hydroxyl radicals (•OH), can cause oxidative damage to lipids, proteins, and DNA, affecting cellular homeostasis and neurodevelopment. These redox alterations are also associated with inflammatory activity at both systemic and brain levels. Overall, these processes impair neuronal and synaptic function, cause changes in dendritic spine density, and affect synaptic pruning. Together, these converging events contribute to the pathophysiology of autism spectrum disorder.

Cellular ROS are generated as inevitable byproducts of mitochondrial oxidative phosphorylation and by various enzymatic reactions, including those catalyzed by NOXs [39]. Superoxide radicals are produced by both Complex I and Complex III of the mitochondrial electron transport chain and are rapidly dismuted by SOD into H_2_O_2_, which is then detoxified by CAT and GPX [19]. These reactions maintain H_2_O_2_ concentrations in the low nanomolar range, which is essential for redox signaling. Mitochondrial dysfunction has been consistently reported in ASD and is indicated by elevated lactate and pyruvate levels, reduced ATP production, and altered oxygen consumption and coupling efficiency. Impaired mitochondrial activity increases vulnerability to metabolic stress and has been linked to immune dysregulation and neuronal impairment, as shown by mitochondrial DNA abnormalities and defects in oxidative metabolic pathways. Mitochondrial inefficiency also affects the function of key antioxidant enzymes, including SOD, CAT, and GPX, leading to increased superoxide leakage, oxidative protein damage, chronic inflammation, and neuronal dysfunction [[40], [41], [42], [43]] (Fig. 1).

Beyond mitochondrial sources, several non-mitochondrial enzymes also contribute to cellular ROS generation. The central nervous system (CNS) is highly dependent on O_2_ and particularly sensitive to changes in its levels [44]. For this reason, NOXs, which are localized to different cellular compartments, such as the plasma membrane and endoplasmic reticulum, are maintained at low levels in resting CNS cells, ensuring a stable and low flux of H_2_O_2_. This steady state allows H_2_O_2_ to oxidize specific cysteine residues in target proteins, modulating the activity of key transcription factors (e.g., NRF2), receptor tyrosine kinases, and ion channels, which in turn promotes neuronal survival and differentiation [45]. However, insults to the CNS, such as those occurring in ASD and associated with environmental factors (e.g., MIA and toxic exposures), neuroinflammation, mitochondrial dysfunction, and genetic factors, may lead to upregulation of NOX activity, increased oxidant production, and consequent oxidative damage [37,38,[46], [47], [48]]. It has also been hypothesized, and partially supported by an observed association between peroxisomal biogenesis and ASD, that disturbances in peroxisomes may play a role in the pathophysiology of ASD. Peroxisomes are oxidative organelles that generate H_2_O_2_ as a byproduct of various metabolic reactions, including fatty acid β-oxidation and amino acid catabolism [[49], [50], [51]]. Recently, the kynurenine pathway, the major pathway of tryptophan catabolism and a key source of nicotinamide adenine dinucleotide (NAD^+^), has gained increasing attention in the context of ASD. Several metabolites associated with the kynurenine pathway possess pro-oxidant properties that may directly contribute to oxidative stress or exacerbate it in susceptible individuals with ASD [52]. Notably, 3-hydroxykynurenine (3-HK) promotes ROS generation through auto-oxidation and interaction with transition metals, while quinolinic acid (QA) induces lipid peroxidation and depletes cellular antioxidants. Notably, 3-HK is a direct precursor to QA within the kynurenine pathway, as it is converted to 3-hydroxyanthranilic acid and subsequently to QA. This biochemical link suggests that elevated 3-HK may contribute to oxidative stress both directly and indirectly, by increasing QA levels and amplifying excitotoxic and oxidative damage [53,54]. It has also been demonstrated that MIA increases 3-HK and ROS levels in both the placenta and fetal brain of animal models, potentially contributing to the development of neuropsychiatric disorders such as ASD [55].

## 3 Oxidative stress biomarkers in ASD

From a clinical standpoint, multiple classes of biomarkers exhibit significant differences compared with neurotypical controls, suggesting the involvement of oxidative stress and impaired antioxidant defenses in ASD. Elevated levels of lipid peroxidation markers, protein oxidation biomarkers, DNA oxidation products, and altered antioxidant enzyme activity have been consistently reported across several clinical studies (Table 1). 8-Iso-prostaglandin F_2_α (8-iso-PGF_2_α), an isoprostane formed through the non-enzymatic peroxidation of arachidonic acid in cell membranes, is recognized as the clinical gold standard for assessing lipid oxidative stress in vivo. Several studies have reported elevated plasma and urinary levels of 8-iso-PGF_2_α in children with ASD [[56], [57], [58], [59]]. Likewise, elevated malondialdehyde (MDA) levels, a product of polyunsaturated fatty acid (PUFA) peroxidation in cell membranes, have been observed in the frontal brain regions of individuals with ASD, as well as in the plasma and serum of children with ASD [18,[60], [61], [62], [63], [64], [65], [66], [67]]. Although few studies have focused on 4-hydroxynonenal (4-HNE), a major aldehyde product of the peroxidation of omega-6 PUFA, including linoleic and arachidonic acids, elevated levels have been reported in individuals with ASD, suggesting its potential as a peripheral biomarker of lipid peroxidation [68,69]. Protein carbonyls, which result from the introduction of carbonyl groups into the protein structure, are among the most widely used biomarkers for assessing protein oxidation. This modification can occur through the direct oxidation of amino acid side chains or reactions with lipid peroxidation products. Elevated carbonyl levels in two proteins involved in the complement system and immunoregulation have been found in the plasma of autistic children [70]. Protein oxidation has also been reported in the cerebellum and other specific brain regions of individuals with ASD by Sajdel-Sulkowska et al., showing increased levels of 3-NT, which is formed by the reaction of superoxide with nitric oxide (NO) on tyrosine residues in proteins [71,72]. Multiple studies have demonstrated elevated levels of NO, nitrite, and peroxynitrite (ONOO^−^) in individuals with ASD. For instance, plasma nitrite concentrations in children with ASD have been reported to be 1.38-fold higher than in controls [73,74]. NO is synthesized by nitric oxide synthase (NOS) using tetrahydrobiopterin (BH_4_) as a cofactor. Under conditions of BH_4_ deficiency, NOS becomes uncoupled, leading to the production of peroxynitrite, a highly reactive nitrogen species (RNS), rather than NO. This uncoupling has been closely linked to mitochondrial dysfunction, a frequently observed feature in ASD [75]. Both NO and peroxynitrite negatively affect mitochondrial function by inhibiting electron transport chain activity, thereby impairing ATP production and enhancing oxidative damage. It has been demonstrated that lymphoblastoid cell lines (LCLs) derived from individuals with ASD exhibit increased vulnerability to NO-induced mitochondrial dysfunction, suggesting that ASD LCLs may have intrinsic deficiencies in mitigating nitrosative stress [76].

**Table 1:** Biomarkers of oxidative stress and antioxidant defense in autism spectrum disorder.

Biomarkers	Sample type	Method	Population	Results	Study
8-iso-PGF_2_α	Urine	ELISA	ASD (n = 21) vs. NT (n = 20), age 5–12	↑ 8-iso-PGF_2_α in ASD	[56]
8-iso-PGF_2_α	Urine	ELISA	ASD (n = 33), NT (n = 29); age 4–17	↑ 8-iso-PGF_2_α in ASD	[59]
8-iso-PGF_2_α	Serum	ELISA	ASD (n = 24) vs. NT (n = 24), age 9–10	↑ 8-iso-PGF_2_α in ASD	[57]
MDA	Plasma	TBARS assay	ASD (n = 30) vs. NT (n = 30), age 3–15	↑ MDA in ASD	[62]
MDA	Plasma	Spectrophotometric	ASD (n = 52) vs. NT (n = 48), age 3–6	↑ MDA in ASD	[18]
MDA	Plasma	TBA assay	ASD (n = 9) vs. NT (n = 2), age 6	↑ MDA in ASD	[61]
MDA	Plasma	ELISA	ASD (n = 30) vs. NT (n = 30), age 3–11	↑ MDA in ASD	[66]
MDA	Plasma	N/A	ASD (n = 19) vs. NT (n = 19), age N/A	↑ MDA in ASD	[63]
MDA	Frontal cortex (postmortem)	Immunofluorescence	ASD (n = 5), dup(15q/ASD) (n = 5) vs. NT (n = 5), age 7–32	↑ MDA in ASD	[60]
MDA	Serum	Not specified	ASD (n = 45) vs. NT (n = 42), age 3–11	↑ MDA in ASD	[65]
MDA	Plasma	TBARS assay	ASD (n = 35) vs. NT (n = 34), age 7–17	↑ MDA in ASD	[67]
4-HNE	Urine	Spectrophotometric	ASD (n = 45) vs. NT (n = 50), age 4–12	↑ 4-HNE in ASD	[68]
4-HNE	Plasma and erythrocyte membrane	Western blot	ASD (n = 20) vs. NT (n = 18), age 4–30	↑ 4-HNE in ASD	[69]
Protein carbonyls (C8A, Igkc)	Plasma	2D-Oxyblot + Western blot	ASD (n = 15) vs. NT (n = 15), age 2–6	↑ protein carbonyls in ASD	[70]
3-NT	Cerebellum (postmortem)	ELISA	ASD (n = 5) vs. NT (n = 3), age 5–32	↑ 3-NT in ASD	[72]
3-NT	Multiple brain regions (postmortem)	ELISA	ASD (n = 2) vs. NT (n = 2), age 8.8–14.6	↑ 3-NT in ASD	[71]
8-oxodG in cfDNA	Plasma (cfDNA)	Immunodot blot	ASD (n = 133) vs. NT (n = 27), age 4–12	↑ 8-oxodG in severe ASD group	[77]
8-oxodG	Serum	ELISA	ASD (n = 60) vs. NT (n = 29), age 3–20	↑ 8-oxodG in ASD	[78]
8-oxo-dG	Lymphocytes	LC-MS-MS	ASD (n = 84), age 3–18	↓ 8-oxo-dG in CC genotype vs. T+ carriers (NRF2 SNP)	[118]
Nitrite, GPX, SOD	Plasma (nitrite in erythrocyte)	Colorimetric assay	ASD (n = 27) vs. NT (n = 30), age 2–13	↑ Nitrite and ↑ GPX in ASD, = SOD	[74]
GSH, GSSG, GSH/GSSG ratio	Plasma	HPLC	ASD (n = 43) vs. NT (n = 41), age 3–10	↓ GSH, ↓ GSH/GSSG, ↑ GSSG in ASD	[80]
GSH, GSSG, GSH/GSSG ratio	Cerebellum (postmortem)	HPLC	ASD (n = 15) vs. NT (n = 12), age N/A	↓ GSH, ↓ GSH/GSSG, ↑ GSSG in ASD	[17]
GSH, GSSG, GSH/GSSG ratio	Plasma	HPLC	ASD (n = 55) vs. NT (n = 44), age 5–16	↓ GSH, ↓ GSH/GSSG, ↑ GSSG in ASD	[81]
GST	Blood	Genotyping	ASD (n = 113), age N/A	GST polymorphisms associated with ASD severity	[82]
SOD, CAT	Erythrocytes	Spectrophotometry	ASD (n = 52) vs. NT (n = 48), age 3–6	↑ SOD and ↑ CAT in ASD	[18]
SOD	Serum	Colorimetric assay	ASD (n = 96) vs. NT (n = 96), age N/A	↓ SOD in ASD	[83]
GSH, GSSG, GSH/GSSG ratio, SOD, CAT, GPX	Plasma (SOD & CAT in erythrocyte)	Spectrophotometry	ASD (n = 30) vs. NT (n = 30), age 3–15	↓ GSH, ↑ GSSG, ↑ SOD, and ↑ GPX in ASD, = CAT	[62]
SOD, CAT	Plasma	Enzyme activity assay	ASD (n = 10) vs. siblings (n = 10), age 4–10	↓ SOD, ↑ CAT, ↓ SOD/CAT ratio in ASD	[19]
SOD, CAT	Erythrocytes	Spectrophotometry	ASD (n = 27) vs. NT (n = 26), age N/A	↑ SOD, ↓ CAT in ASD	[84]

Abbreviations: 8-Iso-prostaglandin F_2_α, 8-iso-PGF_2_α; Enzyme-linked immunosorbent assay, ELISA; Autism spectrum disorder, ASD; Neurotypical, NT; Malondialdehyde, MDA; Thiobarbituric acid reactive substances, TBARS; Thiobarbituric acid, TBA; Not available, N/A; Duplication, dup; 4-Hydroxynonenal, 4-HNE; Complement component 8 alpha chain, C8A; Igkc Immunoglobulin kappa constant region; 3-Nitrotyrosine, 3-NT; 8-oxo-deoxyguanosine, 8-oxodG; cell free DNA, cfDNA; Liquid Chromatography with tandem mass spectrometry, LC-MS-MS; Nuclear factor erythroid 2-related factor 2, NRF2; Single-nucleotide polymorphism, SNP; Glutathione, GSH; Glutathione disulfide GSSG; High-performance liquid chromatography, HPLC; Glutathione S-Transferase, GST; Superoxide Dismutase, SOD; Catalase, CAT; Glutathione peroxidase, GPX.

Recently, emerging evidence suggests that DNA oxidation may play a significant role in the pathogenesis of ASD by influencing gene expression, epigenetic regulation, and neuronal integrity. For example, it has been reported that oxidized cell-free DNA (cfDNA) obtained from the blood of individuals with ASD acts as a stress-signaling factor, triggering a chronic inflammatory process. Moreover, cfDNA from patients with severe ASD was characterized by a high abundance of oxidative modifications, specifically measured as 8-oxodG [77]. Substantial oxidative damage to DNA in individuals with ASD has also been demonstrated in other studies, where serum 8-oxodG levels were found to be significantly higher in autistic children [65,78].

The endogenous antioxidant defense system, in particular GSH, appears to be dysregulated in ASD. GSH can neutralize hydroxyl radicals and hydrogen peroxide through its thiol group but also serves as a cofactor for antioxidant enzymes, such as GPX, which reduces peroxides to water or alcohol, thus protecting cells from oxidative damage. The balance between reduced GSH and its oxidized form GSSG is a marker of oxidative stress, with a higher GSH/GSSG ratio indicating better antioxidant capacity [79]. A large meta-analysis found that children with autism have lower levels of GSH, total glutathione (tGSH), and GSH/GSSG ratio, along with higher GSSG, indicating a shift toward an oxidized redox state [16]. This reduction in glutathione redox capacity has been linked to impaired mitochondrial enzyme activity (e.g., decreased aconitase activity), excess mitochondrial superoxide production, neuroinflammation, immune dysregulation, and oxidative damage to macromolecules [17,80]. Importantly, glutathione redox imbalance in ASD has been shown to correlate with ASD severity. For instance, autistic children have 21 % lower GSH levels and a 49 % higher GSSG/GSH ratio than controls, and these oxidative stress markers were among the biochemical differences associated with more severe language and social impairment [81]. Moreover, specific GST polymorphisms have been linked to more severe ASD symptoms and lower adaptive functioning, suggesting that genetic factors influencing glutathione metabolism may affect the clinical presentation of ASD [82].

Other studies on endogenous antioxidant enzymes, such as SOD and CAT, which are considered potential biomarkers of oxidative stress, have reported conflicting results. SOD and CAT activity has been found to be higher in erythrocytes from individuals with ASD compared to controls, which may reflect a compensatory response to elevated oxidative stress levels [18]. However, a case-control study reported decreased serum SOD levels in ninety-six children with ASD [83]. Interestingly, other findings indicate that serum CAT is elevated in individuals with ASD, while CAT activity in red blood cells (RBCs) is reduced in autistic children. This discrepancy may reflect tissue-specific differences in antioxidant responses, as well as differences in the underlying etiology of ASD and neurodevelopmental stage. Moreover, it has also been observed that the SOD/CAT ratio is often decreased due to altered CAT activity [19,62,84].

## 4 Oxidative and nitrosative stress in synaptic dysfunction and ASD

Oxidative and nitrosative stress are increasingly recognized as key contributors to the pathogenesis of ASD, particularly in the context of synaptic dysfunction. They converge on synaptic processes through multiple mechanisms, including direct protein modifications, mitochondrial impairment, and disrupted neurotransmitter dynamics, which together compromise the fidelity of synaptic signaling. The resulting synaptic impairments provide a mechanistic link between cellular stress and the complex behavioral features associated with ASD [85]. One of the mechanisms through which nitrosative stress affects synaptic function involves posttranslational modifications of proteins. Peroxynitrite, a potent RNS formed by the reaction of NO with superoxide, can nitrate tyrosine residues in proteins, leading to the accumulation of 3-NT. As mentioned above, elevated levels of 3-NT have been reported in individuals with ASD, indicating extensive protein alterations in the brain. These nitrosative modifications interfere with the structure and function of critical synaptic proteins, impairing neurotransmission, synaptic vesicle trafficking, ion channel activity, and postsynaptic receptor clustering [71]. Another important nitrosative mechanism is S-nitrosylation, the covalent attachment of NO to cysteine thiol groups, which alters the structure and function of proteins involved in synaptic plasticity [73,86]. While physiological S-nitrosylation regulates processes, such as glutamate receptor modulation and mitochondrial dynamics, excessive NO levels in ASD affect synaptogenesis as well as the glutamatergic and GABAergic systems in the cortex and the striatum, contributing to ASD-like behavioral features [87]. Moreover, aberrant S-nitrosylation of proteins, such as dynamin-related protein 1 (Drp1), exacerbates mitochondrial fission, impairing energy production and calcium buffering, both essential for synaptic vesicle cycling and neurotransmitter release [[88], [89], [90]].

Peroxynitrite and other RNS damage mitochondrial components, impair oxidative phosphorylation, and promote further production of ROS and RNS, establishing a feedback loop that exacerbates cellular stress. Mitochondrial dysfunction consequently compromises ATP generation, which is essential for synaptic vesicle cycling and efficient neurotransmission. Indeed, this energy deficit has been reported to contribute to synaptic dysfunction in ASD, linking mitochondrial impairment to its neuropathological features [85,91].

Long-term potentiation (LTP), which underlies learning and memory, is particularly vulnerable to oxidative and nitrosative stress [92]. LTP, a process dependent on glutamate receptor activation and calcium signaling, is reduced in ASD due to oxidative inactivation of NMDA receptors and nitrosylation of proteins involved in dendritic spine remodeling [93]. Likewise, excessive ROS and RNS alter the balance between excitatory (glutamatergic) and inhibitory (GABAergic) neurotransmission. Moreover, oxidative damage to glutamate transporters prolongs synaptic glutamate exposure, promoting excitotoxicity [94,95]. Conversely, nitrosative stress reduces the expression of glutamic acid decarboxylase (GAD), the enzyme that converts glutamate to GABA. Accordingly, the reduction in GABA synthesis leads to an imbalance in the excitatory-inhibitory neurotransmitter balance, shifting towards hyperexcitability, a pattern consistently reported in ASD neurobiology [96,97]. These converging redox-driven alterations in synaptic signaling, plasticity, and neurotransmitter balance may collectively contribute to the core neurobehavioral manifestations of ASD, including cognitive inflexibility, sensory hypersensitivity, and social communication deficits.

## 5 NRF2 signaling pathway: an overview

Eukaryotic organisms have evolved sophisticated strategies to counteract oxidative and electrophilic stress resulting from metabolism or xenobiotic exposure. NRF2 is a versatile transcription factor that regulates a broad network of detoxification and antioxidant genes. Human NRF2 consists of seven NRF2–ECH homology domains (Neh1–7). The DLG and ETGE motifs in Neh2 interact with Kelch-like ECH-associated protein 1 (KEAP1), while Neh1 contains a basic-region leucine zipper (bZIP) motif that recognizes AREs in target gene promoters [98,99]. The importance of the NRF2 pathway in cellular homeostasis has led to its highly regulated control, with KEAP1 acting as its main regulator.

**Fig. 2:**
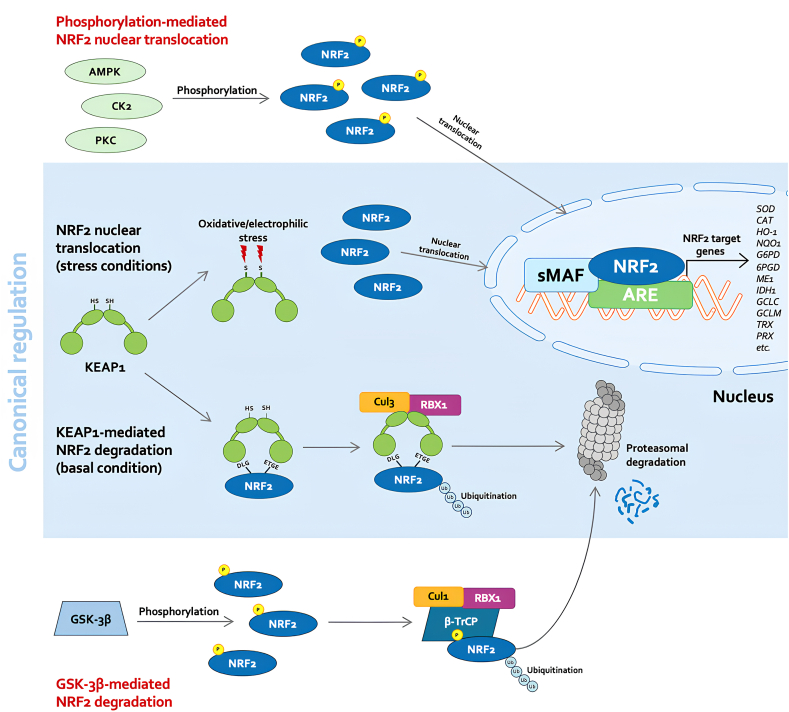
**Canonical and non-canonical regulation of NRF2 stability and activity**. Under homeostatic conditions, NRF2 is constitutively ubiquitinated by the KEAP1–CUL3–RBX1 E3 ligase complex and degraded via the proteasome. Oxidative or electrophilic stress modifies reactive cysteines on KEAP1, impairing NRF2 ubiquitination and allowing its nuclear translocation. Instead of KEAP1, NRF2 can also be regulated through GSK-3β–mediated phosphorylation, which targets NRF2 for β-TrCP–dependent ubiquitination via the CUL1–RBX1 complex. Likewise, phosphorylation by kinases such as AMPK, CK2, and PKC enhances NRF2 stability and promotes its nuclear accumulation. In the nucleus, NRF2 heterodimerizes with small Maf proteins and binds to AREs, inducing transcription of cytoprotective genes including HO-1, NQO1, SOD, CAT, and enzymes involved in glutathione synthesis and the pentose phosphate pathway (e.g., GCLC, GCLM, G6PD, 6PGD, ME1, IDH1). These pathways collectively orchestrate adaptive responses to maintain cellular redox homeostasis.

The “hinge and latch” model describes how structural changes in KEAP1 influence the capture or release of NRF2. Under basal conditions, KEAP1 maintains the correct conformation and forms a complex with Cullin 3 (Cul3) and RING box protein 1 (Rbx1) to bind NRF2 and direct it toward proteasomal degradation [100]. However, in addition to this canonical pathway, other mechanisms also regulate NRF2 (Fig. 2). Glycogen synthase kinase-3β (GSK-3β), for example, phosphorylates NRF2 at specific sites, enhancing its interaction with β-transducin repeat-containing protein (β-TrCP), which in turn forms a complex with S-phase kinase-associated protein 1 (Skp1), Cullin 1 (Cul1), and Rbx1 to ubiquitinate and degrade NRF2 [101,102]. Conversely, protein kinase C (PKC) isoenzymes, casein kinase 2 (CK2), and AMP-activated protein kinase (AMPK) may positively regulate NRF2 activity by phosphorylating distinct sites [103]. The role of KEAP1 is not limited to inhibiting NRF2 activity. It functions as a redox sensor that detects oxidative and electrophilic changes through specific cysteine residues containing thiol groups. Among these, the cysteine residues C151, C273, and C288 are highly reactive and susceptible to covalent modifications by low-level ROS and other electrophiles. When these thiol residues are modified, KEAP1 can no longer interact with the DLG motif, preventing complex formation with Cul3/Rbx1 and, consequently, NRF2 proteasomal degradation [104].

Transport mediated by karyopherin α1 (importin α5) and karyopherin β1 (importin β1) is a critical process that promotes the translocation of newly synthesized NRF2 into the nucleus, where it binds to ARE sequences and induces NRF2-dependent gene expression [105]. ARE motifs are located in several promoter regions encoding antioxidant and detoxifying enzymes, allowing NRF2 to coordinate a wide-ranging cytoprotective response. This NRF2-mediated transcription depends on various cofactors. Small maf proteins interact with NRF2 in recognizing ARE sequences [106]. Likewise, CREB-binding protein (CBP)/p300 and the SUMO-conjugating enzyme UBC9 possess acetyltransferase and SUMOylation activities, respectively, which modulate promoter-specific DNA binding and play a significant role in the regulation of NRF2 activity. These post-translational modifications influence the function of CBP/p300 as transcriptional coactivators, thereby affecting NRF2-mediated gene expression and the cellular antioxidant response [107,108]. Additional transcription factors, such as Jun-D, c-Jun, Jun-B, activating transcription factors 3 and 4 (ATF3, ATF4), receptor-associated coactivator 3 (RAC3), runt-related transcription factor 2 (Runx2), and Yin Yang 1 (YY1), further enhance NRF2-driven transcription [109]. Many genes regulated by NRF2 are critical for restoring redox balance and detoxifying in response to low-to-moderate levels of ROS, particularly SOD, CAT, HO-1, and NQO1. Additionally, NRF2 may increase NADPH levels by regulating glucose-6-phosphate dehydrogenase (*G6PD*), 6-phosphogluconate dehydrogenase (*6PGD*), malic enzyme 1 (*ME1*), and isocitrate dehydrogenase 1 (*IDH1*) [110]. NADPH provides reducing equivalents for numerous antioxidant reactions and supports various cellular processes involved in oxidative stress defense, particularly through GSH. NRF2 also regulates the GSH system by controlling the expression of enzymes required for its synthesis, including glutamate-cysteine ligase (*GCLC* and *GCLM*), GR, GPX, and GSTs. The coordinated regulation of these enzymes maintains glutathione recycling and function, contributing to cellular redox balance and enhancing overall antioxidant capacity [111]. Moreover, NRF2 contributes to redox restoration following oxidative stress by coordinating the expression of Trx, *TXNRD1*, and *PRX* genes [22].

When oxidative stress increases, the crosstalk between the NRF2 and nuclear factor kappa B (NF-κB) pathways plays a crucial role in regulating cellular responses to oxidative stress and inflammation. NF-κB induces the expression of proinflammatory mediators, whereas NRF2 counteracts NF-κB activity both directly and indirectly [112]. These two factors compete for binding to CBP, a cofactor critical for both responses. Accordingly, when one is more active, the other has reduced access to CBP, limiting its ability to induce transcription [113]. By enhancing the expression of antioxidant genes and affecting inflammatory processes, NRF2 mitigates inflammation, at least in part through HO-1 induction. HO-1 catabolizes heme into carbon monoxide, iron, and biliverdin, which is then converted into bilirubin. The NRF2–HO-1 axis can also downregulate the expression of matrix metalloproteinases, either directly or through NF-κB inhibition [114,115]. Under sustained oxidative stress with high levels of ROS, NF-κB can induce additional cytokines and proinflammatory responses, triggering a vicious cycle that exacerbates oxidative stress, inflammation, and cellular damage, which in turn contributes to the impairment of NRF2 activity.

## 6 NRF2 and ASD

The activation of NRF2 by ROS represents a critical adaptive mechanism where low-to-moderate ROS levels stimulate antioxidant defenses, while excessive ROS cause cellular damage [116]. This biphasic response appears impaired in ASD. Indeed, a growing number of clinical and experimental studies indicate significant alterations in NRF2 signaling, suggesting that its dysfunction may contribute to ASD pathophysiology by increasing oxidative stress, inflammation, and metabolic disturbances (Table 2).

**Table 2:** Summary of main findings on NRF2 in autism spectrum disorder.

Study model	Sample type	NRF2 status	Key findings	Study
ASD children	Blood	NRF2 Polymorphism (rs35652124)	↑ pNRF2 with DMF	[118]
ASD children	Granulocytes	↓ NRF2 expression	Impaired redox balance, immunity, and mitochondrial function	[119]
ASD children	Blood (plasma)	↓ NRF2 and KEAP1	↓ GSH, GR, GPX3, GSH/GSSG; ↑ GSSG	[120]
ASD children	Blood	↓ NRF2, ↑ KEAP1, ↓ GSK-3β	Impaired antioxidant pathway with dysregulated KEAP1-GSK-3β	[121]
ASD children	Neutrophils	↓ NRF2 response to DEHP	Impaired NRF2/HO-1 induction by DEHP	[126]
ASD individuals	Frontal cortex	↓ NRF2 expression	Linked to thiol and cobalamin metabolism disruption	[127]
ASD children	Monocytes	↓ NRF2 expression	↑ NF-κB, IL-6, IL-1β, nitrotyrosine	[133]
BTBR mice	Hippocampus	↓ NRF2 in males only	↑ Oxidative stress markers and NOX2, ↓ SOD and GSH in males	[138]
Shank3 KO mice	Cortex, SH-SY5Y cells	↓ NRF2 with Trx1 inhibition	↓ NRF2 and antioxidant genes (NQO1, HO-1)	[20]
Shank3 KO mice	Hippocampus	↓ NRF2 phosphorylation and nuclear translocation	↑ Oxidative stress and inflammation via NRF2 pathway	[139]
Chlorpyrifos-induced ASD (rat)	Hippocampus	↓ NRF2 expression	↑ Oxidative stress, ↑ NF-κB,in astrocyte	[140]
NRF2-KO mice exposed to VPA	Brain	Absent	Increased susceptibility to behavioral and oxidative stress effects of VPA	[141]

Abbreviations: Autism Spectrum Disorder, ASD; Nuclear factor erythroid 2–related factor 2, NRF2; Dimethyl fumarate, DMF; Kelch like ECH-associated protein 1, KEAP1; Reduced glutathione, GSH; Glutathione reductase, GR; Glutathione peroxidase 3, GPX3; Oxidized glutathione, GSSG; Glycogen synthase kinase 3 beta, GSK-3β; Di(2-ethylhexyl) phthalate, DEHP; Heme oxygenase 1, HO-1; Nuclear factor kB, NF-κB; Interleukin 6, IL-6; Interleukin 1 beta, IL-1β; NADPH oxidase 2, NOX2; Superoxide dismutase, SOD; Glutathione, GSH; Thioredoxin 1, Trx1, NADPH quinone oxidoreductase 1, NQO1; Valproic acid, VPA.

As mentioned, the genetic architecture of ASD is complex, involving both rare and common variants that contribute to individual risk. The *NFE2L2* gene, encoding NRF2, is located on human chromosome 2 and exhibits substantial genetic variation, with 583 documented allelic forms, including 18 SNPs linked to disease risk [117]. NRF2 polymorphisms have been identified as part of the broader genetic landscape of ASD, influencing adaptive responses to oxidative and genotoxic stress. The *NFE2L2* rs35652124 polymorphism, selected for its high minor allele frequency (≥15 %), affects NRF2 expression. In a cohort of 179 ASD children, those with the CC genotype exhibited higher levels of phosphorylated NRF2 (pNRF2), an active form of the protein that induces antioxidant genes, in response to dimethyl fumarate (DMF), a pharmacological NRF2 inducer, compared to those with T+ genotypes, indicating that this genetic variation influences NRF2 pathway activation by pharmacological agents. Additionally, the CC genotype was associated with increased DNA damage, demonstrated by elevated H2AX levels, but lower plasma 8-oxodG levels, which may reflect differences in DNA repair mechanisms. Consistent with these findings, CC carriers also exhibited a higher cfDNA-to-nuclease activity ratio, suggesting impaired clearance of damaged DNA. Moreover, a correlation between adaptive genotoxic responses, assessed through H2AX, and NRF2-mediated cytoprotection was observed in T+ carriers but not in CC carriers, indicating a disruption in oxidative stress responses in individuals with the CC genotype. These findings suggest that the *NFE2L2* rs35652124 polymorphism modulates oxidative stress responses in children with ASD, affecting their susceptibility to oxidative damage and pharmacological NRF2 activation [118].

In addition to genetic factors, one of the first studies to demonstrate the critical role of NRF2 downregulation in mitochondrial dysfunction and immune anomalies in ASD was conducted in granulocytes from children with ASD. The authors reported a 45 % reduction in NRF2 transcript levels compared to typically developing controls, correlating with systemic oxidative stress and impaired bioenergetics. Reduced NRF2 compromised cellular antioxidant defenses, leading to increased mitochondrial ROS production. Likewise, elevated mtDNA deletions indicated persistent oxidative damage. Furthermore, mitochondrial oxidative phosphorylation capacity in ASD granulocytes was reduced, with deficits across multiple electron transport chain complexes [119].

A recent pilot study further supports the involvement of oxidative stress in ASD by examining the relationship between NRF2 signaling and glutathione homeostasis in young children. The study reported reduced levels of NRF2 and KEAP1 in children with ASD compared to neurotypical controls, along with lower concentrations of GSH, GR, GPX3, and a decreased GSH/GSSG ratio. These alterations were accompanied by elevated levels of GSSG, indicating increased oxidative stress. Notably, a positive correlation was observed between NRF2, GSH, and GR levels, highlighting the role of NRF2 in regulating antioxidant defenses and maintaining redox balance in ASD [120]. A case-control study investigated NRF2, KEAP1, and GSK-3β expression in children with ASD compared to neurotypical controls. NRF2 levels were lower in the ASD group, suggesting an impaired antioxidant response in these children. Conversely, KEAP1 was significantly elevated, contributing to reduced NRF2 availability. Interestingly, GSK-3β expression was found to be lower in the ASD group [121]. Given that GSK-3β typically inhibits NRF2 via proteasomal degradation, its reduction did not correspond to the expected NRF2 increase, suggesting broader pathway dysregulation. However, it is important to note that findings on GSK-3β in ASD are heterogeneous. While some studies report reduced GSK-3β expression, others have shown increased GSK-3β activity, potentially contributing to oxidative stress, inflammation, and altered neurodevelopment. These discrepancies may reflect differences in brain regions, developmental stages, or ASD subtypes. These findings highlight the complexity of GSK-3β regulation in ASD and its intricate relationship with NRF2 signaling [122,123].

As indicated above, extensive evidence suggests that exposure to environmental toxicants, including prenatal in utero exposure, contributes to ASD development [124,125]. Neutrophils from children with ASD show a dysregulated NRF2 response to di(2-ethylhexyl) phthalate (DEHP) exposure, a class of phthalates used as plasticizers, with an impaired ability to upregulate *HO-1*, a crucial NRF2 target gene involved in mitigating oxidative stress. Conversely, neutrophils from neurotypical children exhibit a robust NRF2/HO-1 response to oxidative insults, such as DEHP exposure, whereas neutrophils from children with ASD have a reduced antioxidant response, suggesting a compromised antioxidant defense mechanism in ASD [126].

Decreased NRF2 expression has been reported in the frontal cortex of individuals with ASD, accompanied by disturbances in thiol and cobalamin (vitamin B12) metabolism, both critical for cellular redox balance [127]. Cobalamin plays a crucial role in the development, differentiation, and function of the CNS and participates in the methionine-homocysteine metabolic pathway [128]. Blood vitamin B12 levels in children with ASD are often lower compared to neurotypical children, leading to impaired homocysteine (HCY) remethylation, elevated HCY concentrations, and DNA hypomethylation, potentially disrupting CNS development [16,129,130]. Moreover, reduced methylcobalamin (MeCbl) and GSH levels correlate with lower NRF2 expression, highlighting the relationship between oxidative stress, metabolic dysfunction, and NRF2 dysregulation in ASD. Notably, decreased NRF2 expression in the frontal cortex of individuals with ASD disrupts cobalamin-dependent sulfur metabolism, resulting in reduced methionine and S-adenosylmethionine (SAM) levels, alongside elevated HCY, indicative of compromised methylation capacity and impaired transsulfuration pathway activity [127]. This interdependence between NRF2-mediated antioxidant responses and cobalamin status may drive metabolic disturbances that exacerbate oxidative stress, contributing to neuropathological processes in ASD.

In addition to its role in maintaining redox balance, NRF2 plays a crucial role in regulating inflammation, which can impair normal brain developmental processes such as neuronal migration, differentiation, and synaptogenesis, leading to alterations in brain connectivity and function that contribute to ASD symptoms [131,132]. Monocytes from children with ASD exhibit decreased NRF2 expression and ARE-binding activity compared to those from neurotypical children, as well as increased inflammatory markers, including NF-κB, interleukin-6 (IL-6), IL-1β, and nitrosative stress markers such as nitrotyrosine [133]. These findings suggest that persistent oxidative and nitrosative stress in monocytes from children with ASD may contribute to NRF2 pathway exhaustion through mechanisms such as negative feedback (e.g., increased degradation or suppression of NRF2), post-translational modifications impairing NRF2 stability or function, and potential epigenetic silencing of the NRF2 gene, leading to impaired antioxidant responses and increased susceptibility to inflammatory damage.

Nitrosative stress, caused by RNS, such as NO and peroxynitrite, can activate the NRF2 signaling pathway. This activation is considered a key cellular response to reduce NO-induced apoptosis and support cell survival. Nitrosative stress activates the NRF2 pathway through oxidation-induced intermolecular disulfide bond formation between KEAP1 molecules, particularly involving Cys151, which alters KEAP1 conformation, inhibits NRF2 ubiquitination, and promotes NRF2 stabilization and nuclear translocation [134]. Moreover, although direct inhibition of the GSK-3/β-TrCP axis by nitrosative stress is not fully established, nitrosative stress can activate upstream signaling pathways (e.g., PI3K/Akt) that inhibit GSK-3, thereby preventing NRF2 degradation and enhancing its stability. Additionally, reactive nitrogen species, such as peroxynitrite, stimulate PI3K/Akt signaling, with Akt phosphorylation promoting NRF2 accumulation by disrupting KEAP1-NRF2 binding [135,136]. Impaired NRF2 function has been observed in several major psychiatric and neurodevelopmental disorders, including depression and ASD, leading to increased nitrosative stress that contributes to the pathophysiology of these conditions [23].

ASD is diagnosed more frequently in males than females, with differences in neurobiological and behavioral features [137]. In a study conducted using the BTBR mouse model of idiopathic autism, male mice exhibited increased repetitive behaviors accompanied by elevated oxidative stress in the hippocampus, as indicated by higher levels of ROS, enhanced expression of pro-oxidant enzymes such as NOX2, and reduced antioxidant defenses characterized by decreased levels of SOD and GSH. These changes were associated with a significant reduction in NRF2 expression, without corresponding alterations in KEAP1 expression, suggesting impaired activation rather than increased degradation of NRF2. Conversely, female BTBR mice showed distinct oxidative stress profiles characterized by increased MDA and elevated *NOX1* expression but maintained an overall more effective antioxidant response without alterations in NRF2 levels [138]. Additionally, mice with *Shank3* mutations, which are used to mimic behaviors similar to those seen in humans with ASD, show a reduction in Trx activity, leading to decreased NRF2 expression and increased oxidative and nitrosative stress. Shank3, a synaptic scaffolding protein, plays a critical role in synapse formation, maturation, and maintenance. Its loss reduces the upregulation of key NRF2-target antioxidant genes, including *NQO1* and *HO-1*, increasing neuronal vulnerability to oxidative damage [20,139]. Other studies using experimental models of ASD have further highlighted the roles of NRF2 and NF-κB as critical components in regulating oxidative stress and neuroinflammation associated with ASD pathology. Chlorpyrifos, an organophosphate pesticide, has been implicated in neurotoxicity, oxidative stress, and neuroinflammation, particularly during early neurodevelopment. In a chlorpyrifos-induced ASD model, excessive glutamate triggered ROS generation, leading to reduced NRF2 expression and increased NF-κB activation, which in turn promoted astrocyte-mediated neuroinflammation and behavioral deficits [140]. Likewise, postnatal exposure to valproic acid (VPA), a widely used anticonvulsant to model ASD-like behaviors in rodents, provided evidence that NRF2-deficient mice are more sensitive to the damaging effects of VPA, showing reduced locomotor activity, deficits in motor coordination, and impaired learning and memory [141].

Overall, these findings highlight a critical role for NRF2 in ASD, with dysregulation occurring at multiple levels, including genetic, metabolic, inflammatory, and environmental factors. In the next section, we provide an overview of how NRF2 dysfunction may be targeted through pharmacological or dietary interventions to improve the oxidative stress response in ASD.

## 7 Antioxidant interventions using NRF2 inducers in ASD

Although ASD is widely recognized as a multifactorial condition, the evidence discussed above demonstrates that its pathophysiology also involves oxidative stress and impaired NRF2 activity. This impairment is influenced not only by tissue- or context-specific factors, but also by genetic polymorphisms, mitochondrial dysfunction, neuroinflammation, environmental exposures, and disruptions in methylation and cobalamin metabolism [127,142]. Nonetheless, reduced NRF2 activity appears to be observed in certain contexts within ASD. Therefore, NRF2 can be targeted to regulate oxidative stress and enhance its activity. Notably, many NRF2 modulators act through hormetic mechanisms, inducing mild oxidative challenges that trigger adaptive cellular defenses and activate NRF2 target genes [143,144]. Several promising NRF2 inducers are currently under investigation, including in preclinical and clinical studies on ASD. There are two classes of NRF2 inducers: cysteine-reactive inducers and protein-protein interaction inhibitors (PPI) [145]. Recent evidence from systematic reviews indicates that numerous antioxidant compounds, including flavonoids, N-acetylcysteine (NAC) and sulforaphane (SFN), many of which are well-known NRF2 inducers targeting specific cysteine residues on KEAP1, may improve oxidative stress biomarkers, promote the expression of NRF2 target genes, and ameliorate behaviors associated with ASD. However, while SFN and NAC have been evaluated in some clinical trials, most studies have been performed in ASD animal models, and in many cases, direct NRF2 measurement was not included. [146,147]. Given the limited translational applicability of ASD experimental models [148,149], we discuss below only antioxidant compounds known as NRF2 inducers that have been tested in clinical trials (Table 3).

**Table 3:** Clinical studies in autism spectrum disorder involving NRF2-inducing antioxidant compounds.

Study Design	Age	Sample size (Intervention/Control)	Compound (Dosage/day)	Duration	Outcomes	Results	Study
Randomized, double-blind, placebo-controlled clinical trial	13–27 yrs	29/15	SFN (50–150 μmol/day)	18 weeks	ABC, SRS, CGI-I	↓ ABC 34 %, ↓ SRS 17 %	[153]
Follow-up case series	13–27 yrs	Follow-up on 26 participants from Singh et al., 2014 trial	SFN (50–150 μmol/day)	Follow-up after 2.5–4 years	Behavioral follow-up (unscored)	Sustained behavioral improvements	[154]
Randomized, double-blind, placebo-controlled clinical trial	3–27 yrs	27/30	SFN (based on BSA)	15 weeks	Behavior, metabolomics	↓ irritability; improved social and gastrointestinal symptoms, redox modulation	[155]
Ex vivo controlled study	N/A	Monocyte samples from ASD children (number not specified)	SFN (in vitro treatment)	N/A	NRF2, cytokines	↑ NRF2, ↓ inflammation	[133]
Randomized, double-blind, placebo-controlled clinical trial	4–12 yrs	30/30	SFN (50 μmol/day ≤45 kg; 100 μmol/day >45 kg) + risperidone	10 weeks	ABC	↓ irritability, ↓ hyperactivity	[156]
Open-label clinical trial	School-aged (age not specified)	15 (no control)	SFN (dose not specified)	12 weeks	ABC, SRS	SRS improved by 9.7 points ABC improved by 7.1 points	[157]
Randomized, double-blind, placebo-controlled, multicenter trial	3–15 yrs	54/54	SFN (dose not specified)	12 weeks	ADOS, SRS, CGI	Significant improvement on clinician-rated scales; not on caregiver	[158]
Randomized, double-blind, placebo-controlled study	3–7 yrs	20/20	SFN (dose not specified)	36 weeks	ADOS-2, SRS-2, ABC	No significant differences between groups on any measures	[159]
Randomized, double-blind, placebo controlled trial	3.1–9.9 yrs	51/51	NAC (500 mg/day)	6 months	SRS, CCC-2, RBS-R Vineland, SCQ, ADOS	No differences between treatment and placebo groups	[163]
Randomized, double-blind, placebo controlled trial	4–12 yrs	16/15	NAC (up to 60 mg/kg/day; max 4200 mg)		CGI-I, ABC, SRS, VABS-II, oxidative stress markers	No differences in CGI-I, ABC, SRS, or VABS-II between groups; GSH ↑ in NAC group	[164]
Double-blind, randomized, placebo-controlled pilot trial	3.2–10.7 yrs	14/15	NAC 900 mg/day → 1800 mg/day → 2700 mg/day	12 weeks	ABC, SRS, RBS-R	Significant improvement in irritability	[165]
Double-blind, placebo-controlled randomized crossover pilot study	5–16 yrs	24	Glutathione or Glutathione + Vitamin C + NAC (weekly)	8 weeks per phase (2x) +1 week wash out	Behavioral measures, plasma glutathione levels	No significant improvements, mild positive trends across all groups	[166]
Randomized, double-blind, placebo-controlled clinical trial	Children and adolescents (age not specified)	20/20	NAC (1200 mg/day) + risperidone	8 weeks	ABC Irritability subscale	Significant reduction in irritability with NAC + risperidone	[167]
Randomized, double-blind, placebo-controlled clinical trial	4–12 yrs	20/20	NAC (600–900 mg/day) risperidone (1–2 mg/day)	10 weeks	ABC-C	Significant reduction in irritability and hyperactivity	[168]

Abbreviations: Sulforaphane, SFN; Aberrant behavior checklist, ABC; Social responsiveness scale, SRS; Clinical global impressions – improvement scale, CGI-I; Body surface area, BSA; Not Applicable, N/A; Nuclear factor erythroid 2–related factor 2, NRF2; Autism diagnostic observation schedule, ADOS; Autism diagnostic observation schedule, second edition, ADOS-2; N-acetyl cysteine, NAC; Children's communication checklist – second edition, CCC-2; RBS-R: Repetitive behavior scale – revised, RBS-R; Social communication questionnaire, SCQ; Vineland adaptive behavior scales, second edition, VABS-II; Glutathione, GSH; Aberrant behavior checklist – community, ABC-C.

Currently, one of the most promising clinical candidates for ASD is SFN, a naturally occurring isothiocyanate found in cruciferous vegetables and known for its ability to modulate oxidative stress and inflammation. SFN is highly reactive due to the electrophilic nature of its isothiocyanate group, which activates NRF2 by modifying KEAP1 Cys151, increases the synthesis of GSH, and enhances the expression of NRF2-target genes such as *NQO1*, Trx, *TXNRD1*, *GPX2*, among others [[150], [151], [152]].

In a seminal randomized, placebo-controlled clinical trial, young individuals with ASD have been administered daily doses of SFN-rich broccoli sprout extract for 18 weeks, showing significant improvements across several behavioral dimensions, including decreased irritability, hyperactivity, stereotypical behaviors, and enhanced social responsiveness [153]. Moreover, findings from a follow-up case series with the same cohort suggest that SFN had beneficial effects during the subsequent three-year follow-up period [154]. However, the same research group, unable to use the previous SFN-rich preparations in a new cohort, reported that SFN led to small but non-statistically significant changes in the clinical outcomes of 45 children with ASD [155]. In monocytes from children with ASD, NRF2 expression and activity are initially impaired, which is linked to increased inflammation and nitrosative stress. However, SFN treatment activates NRF2 by enhancing its nuclear translocation and DNA-binding activity to AREs. This activation counteracts oxidative/nitrosative stress and inflammation by downregulating NFκB signaling and reducing pro-inflammatory cytokines and stress marker [133].

Another clinical study assessed SFN supplementation in combination with risperidone, an antipsychotic demonstrating significant improvements in irritability and hyperactivity scores, as well as a trend toward improvement in stereotypic behaviors in children with ASD [156]. In an open-label study of children with ASD treated with SFN, improvements in social responsiveness have been reported. Urinary metabolomics analysis identified 77 metabolites correlated with symptom changes, clustering into pathways related to oxidative stress, among various other metabolic processes [157]. Interestingly, the largest randomized, double-blind, placebo-controlled trial of SFN for ASD treatment to date, which enrolled 108 children, reported mixed results. While caregiver-rated scales showed no significant difference between the SFN and placebo groups, clinician-rated scales indicated significant improvements with SFN treatment [158]. In another pediatric cohort of ASD patients, no significant clinical improvement has been observed in behavioral outcomes following a 36-week SFN treatment [159]. Overall, despite compelling experimental evidence that SFN modulates NRF2, its clinical efficacy in ASD remains inconsistently supported across trials.

NAC, a mucolytic agent approved by the FDA, is a well-known antioxidant compound that increases GSH levels, protecting against oxidative damage. As an inducer of NRF2 activity, the thiol group of NAC may interact with cysteine residues on KEAP1, leading to NRF2 translocation to the nucleus and activation of ARE-driven genes [[160], [161], [162]]. The effect of NAC on reducing abnormal behaviors in ASD has been evaluated in some clinical trials. One large placebo-controlled trial involving 102 participants found no significant improvements in social responsiveness, communication, or repetitive behaviors with NAC treatment [163]. Another 12-week randomized study assessing NAC for core social impairment in ASD also reported no significant clinical effects, despite confirming an increase in GSH levels [164]. However, a pilot study found that NAC led to significant reductions in irritability [165]. Likewise, a recent trial investigating glutathione, alone or in combination with NAC and vitamin C, found no significant behavioral or biological improvements in children with ASD, further demonstrating the limited clinical utility of NAC and other antioxidant interventions in improving ASD-related behavioral symptoms [166]. However, two randomized, double-blind, placebo-controlled studies demonstrated that adding NAC to risperidone led to significantly greater reductions in irritability and hyperactivity compared to risperidone alone. At the same time, NAC had no impact on core ASD symptoms beyond reducing irritability. These findings suggest that NAC may enhance the therapeutic effects of risperidone in managing behavioral symptoms associated with ASD [167,168].

A meta-analysis further evaluated the efficacy of antioxidant interventions in managing ASD symptoms by analyzing 13 double-blind, randomized clinical trials involving a total of 570 patients with ASD. The analysis assessed eight antioxidant compounds, many of which are well-known NRF2 inducers, including NAC, resveratrol, SFN, folic acid, coenzyme Q10, vitamin C, vitamin D, and omega-3 fatty acids. Although effect sizes were small, the findings indicate that antioxidants improved irritability and communication disturbances, with promising trends for stereotypic behavior and hyperactivity symptoms, suggesting that these interventions may play a role in managing certain symptoms in patients with ASD [146].

## 8 Conclusions

Although our understanding of its causes has improved, ASD remains one of the most complex neurodevelopmental disorders, with a combination of genetic, neurobiological, and environmental factors that is not yet fully understood, contributing to its complex phenotype.

The lack of established biomarkers and pharmacological treatments for ASD is mainly due to the clinical and biological diversity of the condition, as well as persistent challenges in biomarker validation and treatment development. The wide variation in genetic, metabolic, and neuroinflammatory profiles among individuals with ASD complicates the identification of reliable and reproducible biomarkers.

Among the various biochemical and metabolic alterations occurring at both systemic and brain levels, we have discussed how oxidative stress and its associated biomarkers may contribute to the pathophysiology of ASD. Multiple biomarkers of oxidative stress, including elevated lipid peroxides, protein and DNA oxidation products, and deficiencies in antioxidant enzyme activities, have been consistently reported in individuals with ASD. These alterations may be linked to an impaired endogenous antioxidant response, mainly regulated by the transcriptional activity of NRF2, whose role in ASD has been increasingly recognized and reported to be dysregulated in experimental and clinical studies. Accordingly, pharmacological and dietary strategies using antioxidant compounds, such as SFN and NAC, which are known to induce NRF2, represent a rational approach to restoring its activity in ASD. However, despite some promising results, current evidence remains inconclusive about their clinical efficacy. Moreover, the majority of clinical trials conducted so far have only focused on measuring behavioral outcomes. Future studies evaluating antioxidant interventions in ASD should include measurements of oxidative stress biomarkers, direct assessments of NRF2 activity, and examination of how these interventions influence NRF2-target genes in ASD populations.

## CRediT authorship contribution statement

**Sergio Davinelli:** Writing – review & editing, Writing – original draft, Investigation, Data curation, Conceptualization. **Alessandro Medoro:** Writing – original draft, Visualization, Resources, Formal analysis. **Martina Siracusano:** Conceptualization. **Rosa Savino:** Writing – original draft, Conceptualization. **Luciano Saso:** Writing – review & editing, Conceptualization. **Giovanni Scapagnini:** Writing – review & editing, Supervision, Conceptualization. **Luigi Mazzone:** Writing – review & editing, Supervision, Conceptualization.

## Ethical approval

Not applicable.

## Funding

No source of funding is associated with this article.

## Declaration of competing interest

The authors whose names are listed immediately below certify that they have NO affiliations with or involvement in any organization or entity with any financial interest (such as honoraria; educational grants; participation in speakers’ bureaus; membership, employment, consultancies, stock ownership, or other equity interest; and expert testimony or patent-licensing arrangements), or non-financial interest (such as personal or professional relationships, affiliations, knowledge or beliefs) in the subject matter or materials discussed in this manuscript.

## Data Availability

No data was used for the research described in the article.
